# PD90 Use Of Medicinal Herbs In Natura By Pregnant Women In The Amazon Region

**DOI:** 10.1017/S0266462324003374

**Published:** 2025-01-07

**Authors:** Celsa da Silva Moura Souza, Erika Barbosa Camargo, Rachel Riera, Regismeire Viana Lima, Edson de Oliveira Andrade, Ana Pilar Betrán Betrán, Maria Regina Torloni

## Abstract

**Introduction:**

There are few studies on the use medicinal herbs by pregnant women in Brazil, even though there is a wealth of knowledge about medicinal herbs among Brazilians of Indigenous, African, and European ancestry. The aim of this study was to assess the prevalence and type of herbs used by pregnant women living in the Amazon region.

**Methods:**

This was a cross-sectional study conducted with 811 pregnant women attending 10 public antenatal clinics in Manaus, Amazonas state, Brazil. The consumption of medicinal herbs was assessed through individual 24-hour dietary recall.

**Results:**

A total of 811 women in their second trimester (16 to 20 weeks) of pregnancy were included and 69 (8.5%) reported that they used herbs to make teas. There was a significant difference between users and non-users of medicinal teas, with a higher proportion of overweight women in the group that used teas (46.4% versus 31.9%; p=0.005). Nearly half (47.8%) of those who used medicinal teas consumed herbs with sedative effects, 23 percent consumed herbs for the relief of urinary tract symptoms, and 13 percent used herbs with digestive properties. Most women reported using natural herbs from their own gardens.

**Conclusions:**

Approximately 10 percent of Brazilian women in the Amazon region consumed medicinal herbs to alleviate common symptoms of pregnancy. The most frequently used plants had sedative, urinary tract, or gastrointestinal effects. Most plants were obtained in natura from local gardens. Many of these plants have known adverse effects and their use is contraindicated during pregnancy.

